# Conic Sampling: An Efficient Method for Solving Linear and Quadratic Programming by Randomly Linking Constraints within the Interior

**DOI:** 10.1371/journal.pone.0043706

**Published:** 2012-08-27

**Authors:** Oliver Serang

**Affiliations:** 1 Department of Neurobiology, Harvard Medical School, Boston, Massachusetts, United States of America; 2 Department of Pathology, Boston Children’s Hospital, Boston, Massachusetts, United States of America; Université de Nantes, France

## Abstract

Linear programming (LP) problems are commonly used in analysis and resource allocation, frequently surfacing as approximations to more difficult problems. Existing approaches to LP have been dominated by a small group of methods, and randomized algorithms have not enjoyed popularity in practice. This paper introduces a novel randomized method of solving LP problems by moving along the facets and within the interior of the polytope along rays randomly sampled from the polyhedral cones defined by the bounding constraints. This *conic sampling* method is then applied to randomly sampled LPs, and its runtime performance is shown to compare favorably to the simplex and primal affine-scaling algorithms, especially on polytopes with certain characteristics. The conic sampling method is then adapted and applied to solve a certain quadratic program, which compute a projection onto a polytope; the proposed method is shown to outperform the proprietary software Mathematica on large, sparse QP problems constructed from mass spectometry-based proteomics.

## Introduction

Linear programming involves optimizing a linear objective function subject to a collection of linear constraints. LP problems are frequently encountered throughout many disciplines, both on their own and as approximations to more complex problems. Linear programming has recently been applied to image reconstruction [1], [2], modeling Markov decision processes [3], and graphical models [4], [5].

Formally, LP requires optimizing an 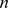 dimensional linear function 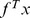 over a feasible region defined by 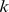 affine inequality constraints 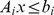. Each row of the 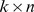 matrix 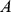, along with the corresponding element in the column vector 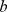, defines a single halfspace, and the feasible region, denoted 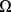, is composed of the intersection of these halfspaces. Thus, any LP problem can be stated as follows:
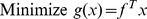


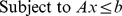



The solution to the LP problem consists of a point 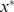 with minimal 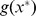.

Finding a feasible point 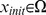 can itself be written as a linear program that maximizes feasibility (this is called a “two phase” approach). Alternately, feasible points can be found during optimization by creating a trivially feasible problem with augmented slack variables 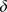, and simultaneously minimizing 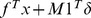. If 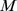 is a large enough constant, the penalty 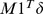 will be driven to 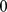 at an optimum (known as the “Big M” method) [6].

### Simplex Methods

The first practical algorithm for solving LP problems, the simplex algorithm [7], was described in 1947. This algorithm embeds the feasible region into a simplex, and then takes steps along vertices on the simplex that decrease the objective function. These steps correspond to movement along the edges of the feasible region, by which one bounding constraint is exchanged for another. When several possible adjacent vertices allow a decrease in the objective value (as is frequently the case), then a pivot rule is used to resolve which will be taken. The simplex algorithm has been shown to have worst-case exponential behavior on certain problems [8] but is efficient in practice, and is still a popular method for solving linear programs. Randomized simplex algorithms, which employ stochastic pivot rules, have been shown to evade exponential behavior [9], but in practice tend to perform worse than deterministic variants. Pseudocode for the steepest-edge and randomized simplex methods implemented for comparison are provided in Algorithm 0, with subroutines as Algorithms 0–0. The simplex variant described and used in this manuscript requires the point 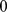 to be in the feasible region; however more sophisticated simplex methods, (*e.g.* the parametric self-dual simplex method [6]) operate using the same basic motivation, but can be used to solve LPs that are not trivially feasible (by implicitly transforming the LP using a method similarly motivated to the Big M method described above, thus manipulating the objective value and the feasibility). These simplex variants can also be used with stochastic pivot rules, and can alternate between primal and dual steps.

**Table:**
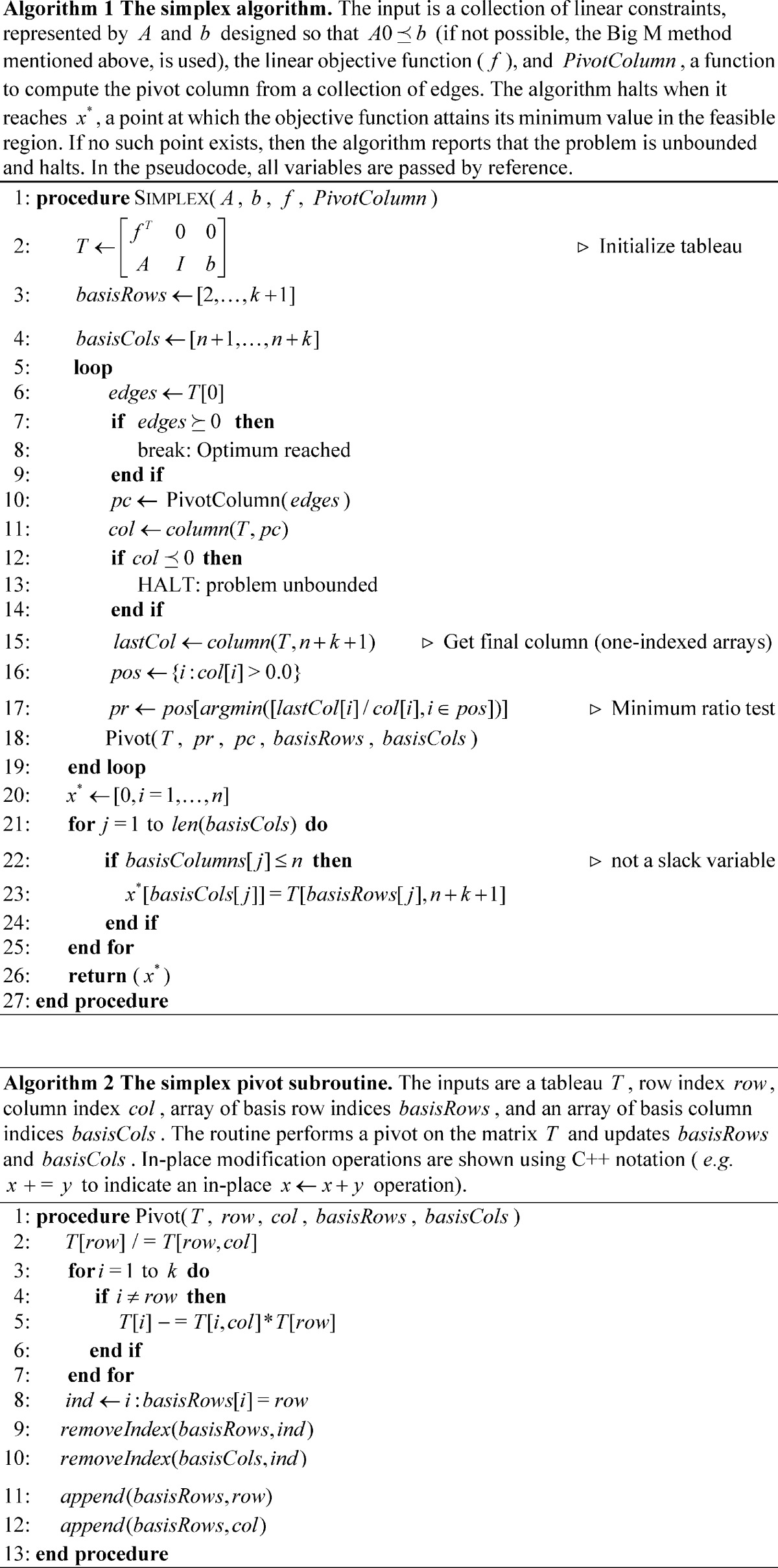

**Table:**
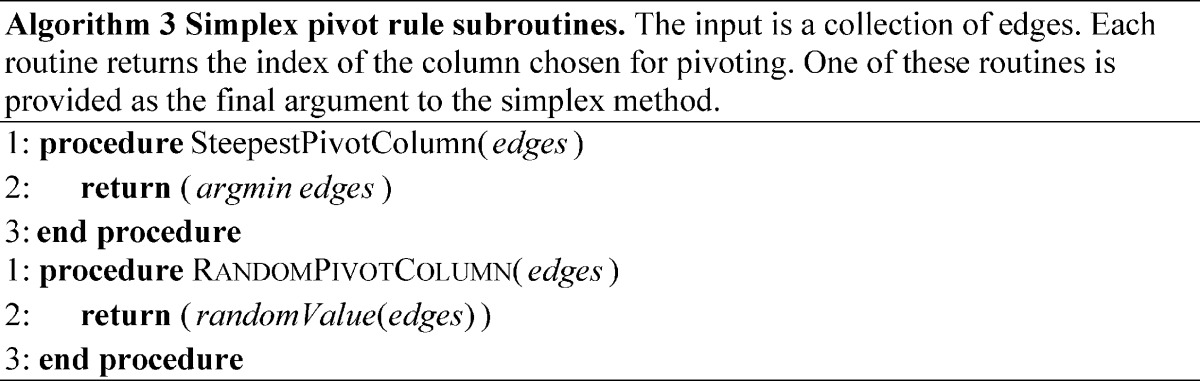

### Generalizations of Simplex Methods

Other geometric methods share similarities to simplex methods and move along the convex hull of the polytope; however, these methods are not restricted to moving along vertices, and so they can be viewed as generalizations of simplex approaches. One such approach is the geometrically motivated gravity descent method [10], which simulates the descent of a very small (radius 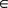) sphere of “mercury” to the minimum of the polytope. As the sphere descends, the walls of constraints it encounters create a reciprocal force, essentially projecting the objective vector to glide along the facets of the polytope. At each iteration, finding the new steepest direction requires solving a small quadratic program (QP) on the set of bounding “active” constraints. Aside from a few subtleties (*e.g.* progressively decreasing the radius of the sphere if it becomes stuck in the vee of two very close facets), the method proceeds in its QP-based descent until the objective value cannot be decreased (as shown by the QP solution).

### Interior Point Methods

In contrast with simplex methods, which traverse adjacent vertices of the polytope, interior point methods remain in the strict interior and asymptotically approach a solution in an iterative manner. Interior point methods terminate once the current solution reaches a predefined precision, and then may optionally use other methods to descend to the nearest vertex and reach an exact solution.

The ellipsoid method was the first algorithm proven to solve the LP to a predetermined precision in a polynomial number of steps [11]. The algorithm successively finds the ellipsoid of minimal volume that contains the intersection of the feasible region and the halfspace requiring the objective value to not increase. In each iteration, a step is taken to the center of the containing ellipsoid, ensuring an exponential decay in the volume of ellipsoids in the series. Although the algorithm converges close to an optimal solution in polynomial time, in practice it is not competitive with the simplex algorithm.

The advent of Karmarkar’s algorithm for LP marked a shift in focus from simplex-based algorithms to interior point methods [12], [13], as well as their primal-dual adaptations [14]. Karmarkar’s algorithm is guaranteed to solve LP problems in polynomial time, asymptotically converging to a desired precision; however, unlike the ellipsoid algorithm, variants of Karmarkar’s interior point method can be fast in practice. The method applies a logarithmic barrier function in lieu of constraints, and takes steps to simultaneously maximize the feasibility and the optimality. A simplified version, the primal affine-scaling method [15], [16], has worst-case exponential behavior but is practically efficient, especially for large, highly constrained LPs. The primal affine-scaling method repeatedly takes steps in the direction of steepest feasible improvement to the objective. It does so in each iteration by inscribing an ellipsoid into the constaints limiting the local feasible region, optimizes the objective function over the hull of the ellipsoid, and finally then takes a step in the direction of that optimum. The ellipsoid is constructed in a manner that scales the space to afford an equal slack to all nearby constraints; this scaling prevents a single nearby constraint from strongly influencing the direction chosen [6]. Pseudocode for the affine-scaling method implemented for comparison is provided in Algorithm 4. Note that sparse vector implementations have low overhead for products between matrices and diagonal matrices, with runtime similar to matrix-vector products.

**Table:**
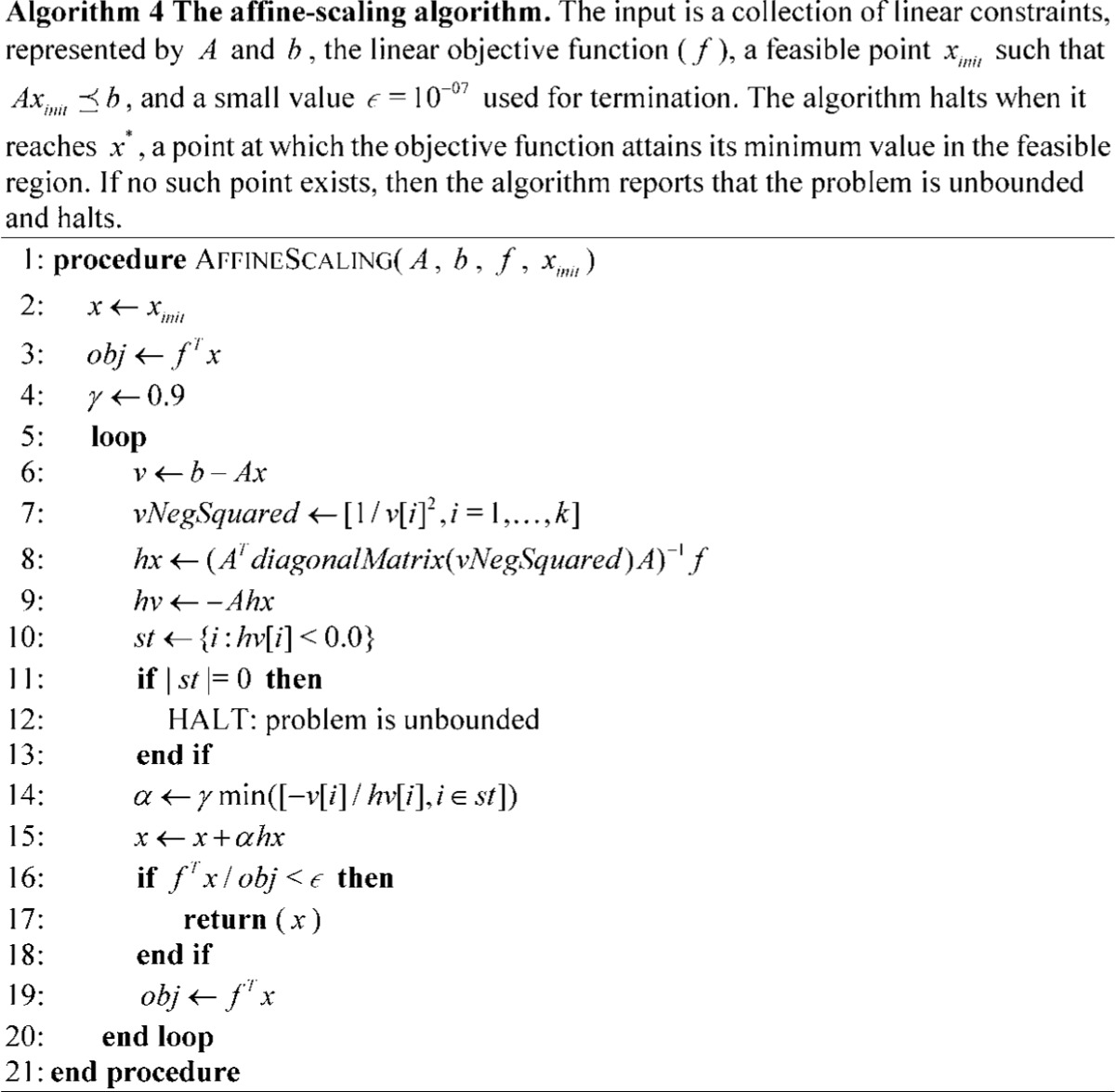

### Additional Randomized Methods

Following the advent and success of the randomized simplex pivot rules, other stochastic algorithms emerged for solving LP. Seidel’s algorithm algorithm randomly downsamples from the set of all constraints [17]. The subproblems will either yield feasible, optimal solutions, or, if not, will indicate that at least one removed constraint bounds the optimum 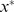. In this manner, the algorithm winnows down set of extreme (*i.e.* important, bounding) constraints. The Matousek/Sharir/Welzl algorithm uses a similar approach, but utilizes further information from the subspace spanned by the basis of currently known extreme constraints [18], thus establishing a new subexponential bound for LP. These algorithms show great promise for future use for have uncovered novel theoretical knowledge of polytopes and the LP problem, but have not yet enjoyed the broad success of simplex methods and interior point methods in practical application.

### A Novel Method that Randomly Links Vertices within the Interior

The simplex algorithm and interior point methods are among the most commonly applied algorithms for LP, due to their simplicity of implementation and their efficiency. Randomized variants of these algorithms are generally thought to perform inefficiently relative to deterministic algorithms. Hence, randomized algorithms are usually only mentioned in the context of avoiding exponential average time performance on pathological problems and are unpopular in practice.

This manuscript describes a simple, geometrically motivated algorithm for LP that randomly samples from the interior of the feasible region in a manner that randomly selects from the set of superior vertices. This stochastic optimization algorithm, named *conic sampling*, is both simple and efficient. For LPs with certain characteristics, the conic sampling algorithm is demonstrated to roughly match or exceed the efficiency of the simplex and primal affine-scaling algorithms, particularly for highly constrained, sparse problems.

## Methods

The proposed algorithm descends to a vertex by taking steps through the interior and projecting orthogonal to constraint rows 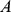 that are encountered. Once a vertex is reached, the algorithm randomly samples from the cone made by these accumulated constraints, finding a direction that improves the objective function and satisfies the accumulated constraints. The algorithm terminates if no direction exists that would satisfy the constraints and improve the objective function.

**Figure 1:**
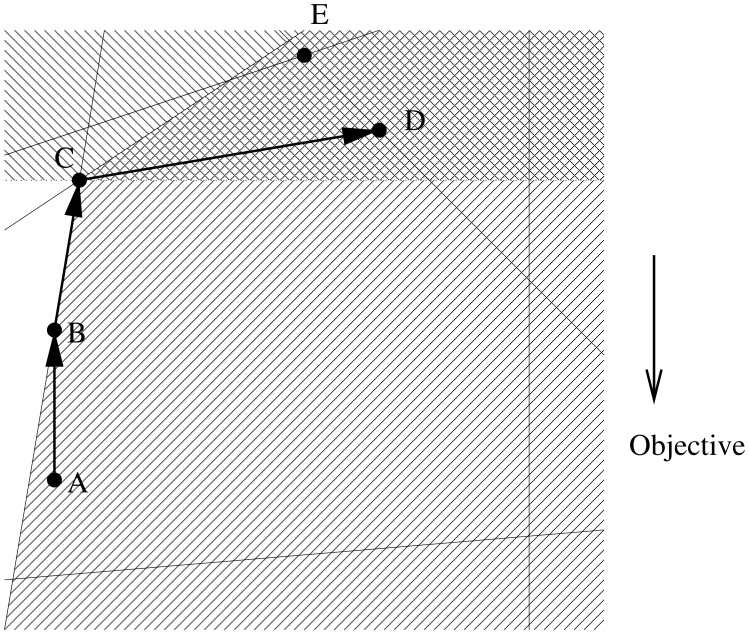
Illustration of conic sampling in two dimensions. The algorithm begins at point A, follows the objective function until it encounters a constraint at B, and then proceeds along the edge of the feasible region to C. At C, the algorithm randomly samples a ray from the cone produced by the intersection of halfspaces defined by improvement on the objective and the polyhedral cone defined by the active constraints. In the figure, this cone is indicated by overlapping shading. Following the sampled ray leads to D. The algorithm continues in a similar fashion, descending to fixation at the minimum point E in the following iteration.

The two-dimensional example in Figure 1 illustrates the basic idea of the conic sampling method applied to an LP problem. Beginning at point A, the algorithm proceeds in a stepwise fashion through points B, C and D. Each step brings the algorithm closer to convergence at point E. In general, the algorithm follows the vector that minimizes the objective function while obeying all of the currently active constraints. Such a move will typically involve traversal across the facet of the polytope that encloses the feasible region. In this process, the procedure will sometimes encounter a vertex, such as C, in which direct movement in the direction decreasing the objective function is not possible.

When a vertex is reached, the set of halfspaces defined by the active constraints, intersected with the halfspace corresponding to non-decreasing objective function, yields a cone of possible legal moves. As the name implies, the conic sampling algorithm randomly selects a ray from within this cone and advances in the selected direction until a new constraint is encountered. Note that, in some degenerate cases, the sampled ray will yield a move of length zero. In this situation, a new ray is sampled until a non-zero move can be made. A pseudocode description of the procedure is given as Algorithm 5, with subroutines as Algorithms 6–8.

The motivation for the conic sampling algorithm is that, given the set of vertices of the polytope in a total ordering by objective value (vertices with equal objective value are never visited sequentially, and so vertices with identical objective can be ignored)
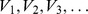


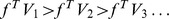



at each iteration starting from vertex 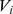, the algorithm will sample from the vertices 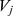 such that 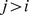. If this sampling is performed uniformly over the remaining candidate vertices then, on average, at each iteration half of the vertices will be eliminated. Although the sampling performed by the conic sampling algorithm is not necessarily uniform, it seems to be close to uniform in practice for certain polytopes. In general, one large advance that occurs at an early iteration early will winnow out many candidates for the next iteration, increasing the chance of choosing the optimum.

The random forward direction subroutine finds the spanning vectors of the cone made by a basis of bounding (also called “active”) constraints and then generates a random conic combination of these vectors that lies in the positive halfspace of 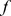. For the cone 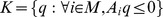, then it has spanning vectors 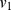, 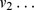, such that 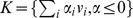. These spanning vectors can be found by projecting every constraint orthogonal to every other constraint. If no vector in the cone can improve the objective, then optimality can be shown. This is similar to trying the 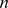 different pivots using the basis 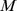 in the simplex algorithm; however, instead of taking the best ray as chosen by some pivot rule, the candidate rays are combined so that the resulting direction is not necessarily restricted to an edge of the polytope.

**Table:**
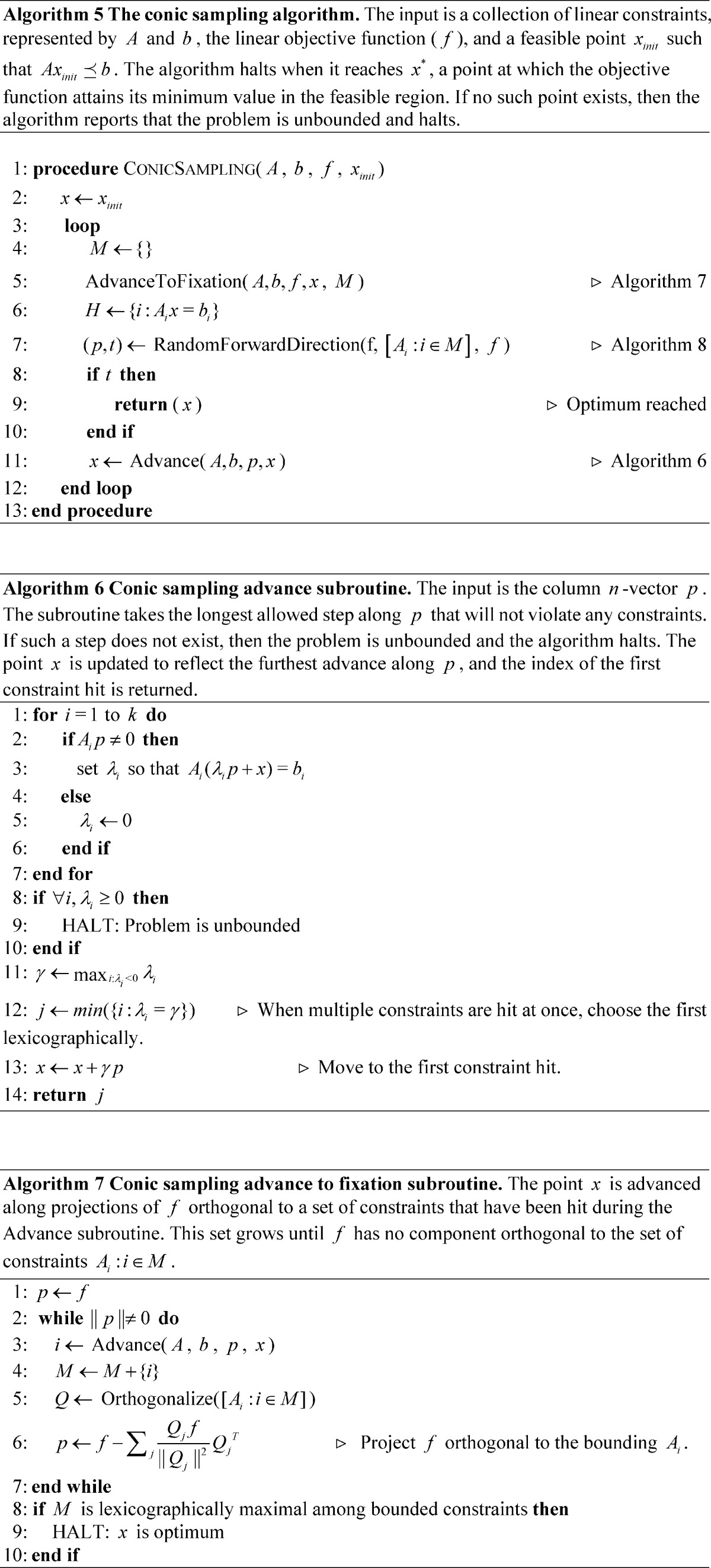

**Table:**
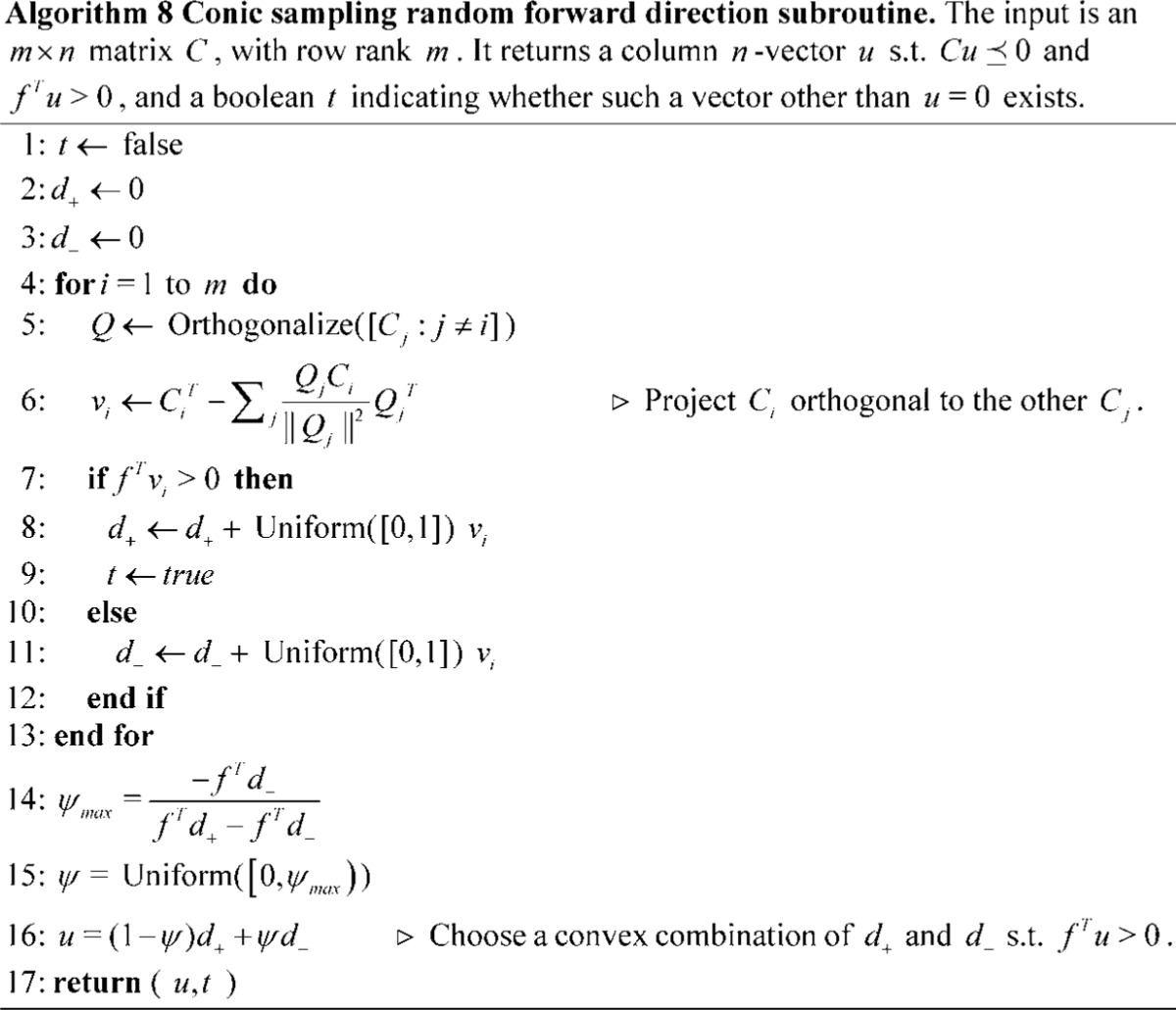

### Proof of Convergence and Optimality

In the following, it is proven that the AdvanceToFixation subroutine terminates and that the ConicSampling algorithm terminates only when optimal. Furthermore, the ConicSampling algorithm is guaranteed to terminate, because the algorithm will advance to an improved vertex with each iteration.

#### Lemma 1

*The* AdvanceToFixation *subroutine terminates in no more than 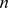 iterations.*

*Proof.* Each iteration calls the Advance subroutine, which must either halt the algorithm or add an element to the set 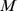. Because 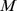 begins empty, inductively assume that the set of vectors 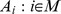 is initially linearly independent. The direction 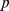 is orthogonalized to these vectors. If the Advance subroutine adds an element 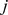 to 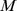, then 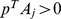. Therefore, 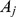 is not a linear combination of the current set of vectors 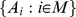, to which 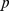 is orthogonal. Thus the set of vectors must be linearly independent, and can therefore contain no more than 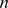 vectors.

#### Lemma 2

*If the algorithm halts, then* 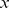 *attains an optimum objective value.*

*Proof.*

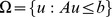


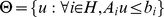


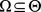



Because 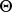 is convex,
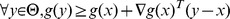



The set 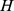 denotes constraints that support 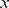:
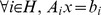


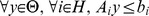


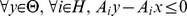


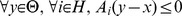



Then for any value of 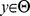, 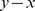 is in a polar cone.
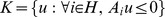


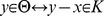



The algorithm terminates when.
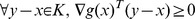


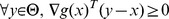


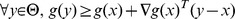


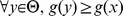



And finally, since 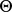 is a superset of 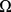,
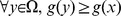



On nondegenerate problems (problems where each vertex has the minimum number of bounding constraints), the method is guaranteed to advance to a new vertex point with each iteration; the number of vertices is, at most, exponential with 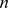.

Degenerate problems are slightly trickier; there it is sufficient to guarantee that the set of constraints bounding a particular vertex are added to 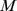 in lexicographic order. By visiting them in this order, the method is guaranteed to add every linearly independent combination of bounding constraints to 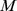; if the vertex is not the optimum, one of these combinations is guaranteed to have a non-empty polyhedral cone and advance. This is trivially observed via Bland’s anticycling pivot rule [19], which is guaranteed to find an edge advancing from any suboptimal vertex (and thus result in at least one non-empty polyhedral cone for sampling). Thus, in the worst case, the algorithm will behave as a less efficient version of the simplex method using Bland’s pivot rule.

### Adaptation to Quadratic Programming

Because the conic sampling algorithm can occupy polytope vertices, facets, and the interior, it can be applied to quadratic programs (QPs), whereas the simplex methods, only occupying vertices, would not be not appropriate. In particular, the method has great potential for efficiently finding the projection of a vector 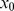 onto a polytope or polyhedral cone:
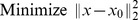


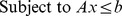



Projections onto polytopes prove a useful exemplar subclass of quadratic programs, because they do not require conjugate gradient descent and can be trivially adapted from the existing algorithm. The modified advance subroutine, which has few changes, given in Algorithm 0, has two main changes: First, the initial local gradient (denoted 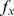 for simplicity, in lieu of all instances of 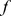 above) is initialized as 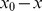 (and re-evaluated each time 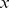 changes). Second, the algorithm does not advance to the limiting constraint if the projection along the free axis 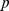 lies inside the polytope.

**Table:**
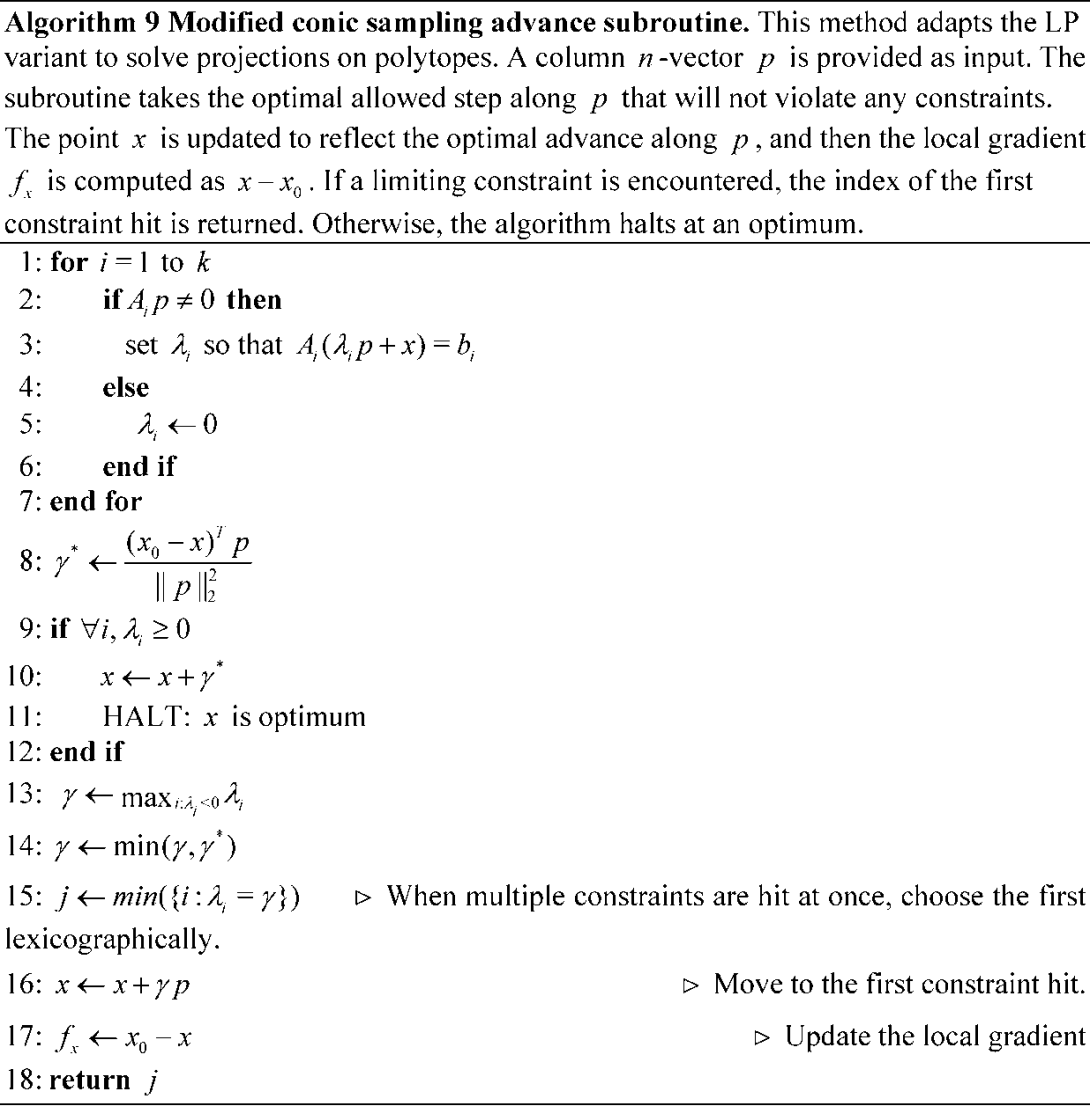

### Proof of Convergence and Optimality for Projection onto Polytopes

Using the above proof of optimality for the LP conic sampling algorithm, it is straightforward to show that the slightly modified algorithm converges to an optimum.

#### Lemma 3

*The modified* ConicSampling *algorithm terminates.*

*Proof.* As before, the algorithm always improves the objective value with each iteration (in the case of degenerate constraints, the anticycling rule is used once again). There are finitely many linearly independent sets of constraints to reach with each iteration, and so the algorithm must terminate.

#### Lemma 4

*The algorithm always finds an optimum in finitely many steps.*

*Proof.* The algorithm has two cases in which it halts (projections cannot be unbounded because the minimum distance is at most 0): The first case is identical to the LP version, where the algorithm halts when the intersection of halfspaces formed by the local gradient 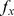 and adjacent constraints is empty. The second case occurs when the projection of 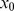 on the set of active constraints (in the modified advance subroutine) lies in the feasible region 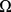; if this is the case, then the projection onto a superset more restrictive feasible region is itself feasible, indicating optimality by definition. Note that this third case of optimality would not be sufficient to demonstrate optimality for general QPs, (but slower conjugate gradient descent can be used).

### Implementation

The conic sampling algorithm, in addition to the simplex method and primal affine-scaling method, are implemented by using a vector library written in C++ (code freely available upon request). Although these implementations are not necessarily expected to be scale competitively on large problems (due to both the efficiency and numeric stability of the implementations), their runtimes should have the same order runtime as more sophisticated variants. In these simulations, proportionality constants should not bias one algorithm over others, because they all designed and optimized with the same vector code base.

Throughout the code, numerical comparisons, particularly when comparing values to zero, were performed using a very small tolerance 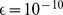. This increases the stability when values deviate from zero due to accumulated numerical error.

### Sparse Vector Math

Methods are compared using a shared code base, and verified using two separate vector data structures appropriate for problems of different size. The first vector data structure, which is most appropriate for small problems (and, hence is used for the preliminary runtime analysis), is a dual sparse-dense data structure. The dual sparse-dense vector data structure indexes the nonzero indices, but also stores the entire vector (zeros included) in a contiguous block to prevent copying (*e.g.* for instance during an insertion of a value into the middle of a vector), and permit efficient random access. Although storing the entire vector as a contiguous block substantially increases the space requirement, it results in very fast in-place vector operations, particularly products between very sparse and very dense vectors. It is worth noting that, aside from loading the matrix 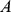 (necessarily performed by all algorithms), the simplex tableau is operated on in a sparse manner, and so entries with values of approximately zero do not influence the runtime).

The second vector library, which only stores the nonzero indices and their values, is less efficient on smaller problems (due to slower in-place operations and slower vector products as described above), but can be applied to much larger problems, where the dual sparse-dense vector data structure become far too memory intensive. This vector library is used to benchmark the algorithms on highly sparse LPs where 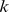 is far larger than 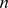.

All algorithms substantially exploit sparsity in 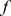, 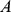, and 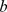. The sparse simplex implementation stores the tableau in a data structure of sparse vectors for each row. More complex variants of the sparse simplex method implemented for these experiments (*e.g.* the Forrest-Tomlin method and Reid’s modified Bartels-Golub method [20], [21]) may have increased numeric stability, but their complexity introduces an increased risk of overhead that may also unfairly penalize the simplex method.

The Orthogonalize routine, likewise, operates using a matrix comprised of an array of sparse vectors. It takes 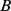 as input and returns a matrix 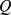 such that rowspan 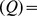 rowspan(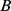) and the rows of 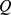 are orthogonal. In practice, this computation can be accomplished efficiently using modified Gram-Schmidt; adding an individual row to 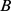 and recomputing 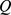 can be performed in 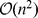 by exploiting the preexisting orthogonality and only adjusting the newly inserted row. Similar considerations have been made when orthogonalizing in the RandomForwardDirection subroutine, where overlapping sets of vectors are repeatedly orthogonalized. Sherman-Morrison updating and sparse LU updating could be used alternatively to compute the projections with the same result, and, possibly, with greater numeric precision. Regardless, more efficiency would be possible by preferrentially ordering the rows so that more sparse rows are on the top, thus preserving their sparsity.

## Results

All runtimes were taken on the same computer using UNIX user time and were programmed using the same code base. Polytopes were chosen so that the feasible region includes 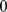 in order to not unfairly penalize the simplex method. C++ programs were compiled with gcc-4.7 using -O3 optimizations.

### Preliminary Runtime Analysis on Random LPs

The runtime of the conic sampling algorithm was compared to the simplex and primal affine-scaling algorithms. The simplex method was implemented to employ different pivot rules: steepest edge and random edge (Algorithm 3). Algorithm runtimes are compared by using the same sparse vector code base and shared common functions (for these preliminary experiments, dual sparse-dense vectors were used, due to their highly efficient in-place access and product operations).

The algorithms were run on a set of randomly generated linear programs, which were made by uniformly (in 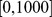) sampling the values of respective rows and values of of 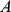 and 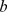 such that 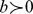, ensuring the feasible region trivially contained 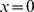. Additional constraints require non-negativity of all elements of 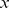 (these constraints are not added to the simplex methods, because they would not influence the result and would increase the simplex runtimes). Each element of the objective vector was likewise chosen uniformly.

**Figure 2:**
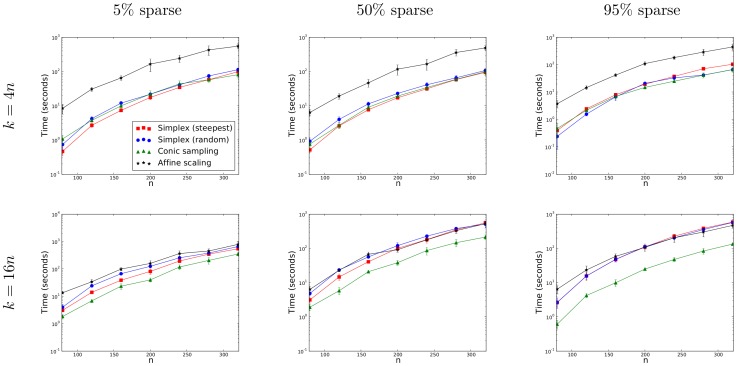
Preliminary runtime analysis on random LPs. The simplex and affine-scaling algorithms are timed against the conic sampling algorithm on random LP problems of varying dimension 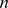 and number of constraints 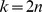 and 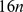. For each 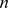 and 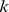, five LP problems were generated. Each panel figure plots the mean runtime as a function of 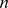. Error bars indicate the minimum and maximum runtimes. Dual sparse-dense vector data structures were used.

The runtimes for different sizes of problems with varying levels of sparsity (*i.e.* varying percents of elements in 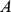, 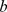, and 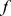 equal to zero) are shown in Figure 2. The 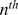 dimension was *not* randomly set to zero so that no zero-norm constraints would be selected. LPs with a high ratio of 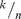 and a high level of sparsity are particularly efficient with conic sampling. Although by no means comprehensive, these random LPs demonstrate the existence of polytopes for which the conic sampling method reliably outperforms simplex variants and affine-scaling.

### Runtimes on Highly Sparse, Random LPs with Varying Numbers of Constraints

Figure 2 illustrates that the greatest performance benefit is achieved on problems with many constraints and a high degree of sparsity. For this reason, the runtime of the conic sampling algorithm was compared to the simplex and primal affine-scaling algorithms on highly sparse (*i.e.* with 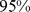 zero values) polytopes with 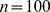 (chosen small enough so that it is practical for the number of constraints 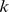 to dwarf 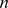) and variable numbers of constraints 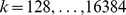. On these LPs, fully sparse vector data structures were used (the dual sparse-dense vector data structure used far too much memory).

**Figure 3:**
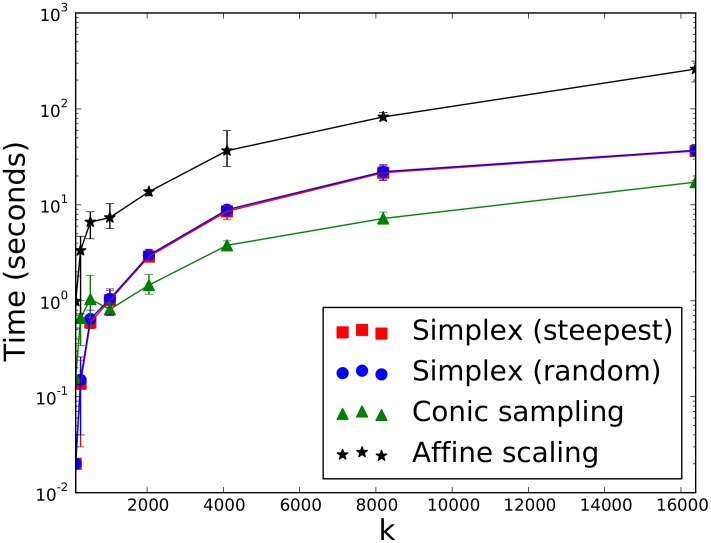
Runtimes on highly sparse, random LPs with varying numbers of constraints. The simplex and affine-scaling algorithms were timed against the conic sampling algorithm on random LP problems with *n* = 100, with 95% sparsity, and with number of constraints *k* = 128,…,16384. For each *k*, three problems were generated and timed with all algorithms. Error bars indicate minimum and maximum runtimes. Fully sparse vector data structures were used.

Figure 3 shows the improvement of the conic sampling method over the affine-scaling and simplex methods on highly constrained, sparse problems.

### Application to QPs from Computational Proteomics

Lastly we demonstrate the efficiency of the modified conic sampling method (*i.e.* the slight modification described above which adapts the method for computing projections on polytopes). We apply this method to polytope projections taken from computational biology. The particular QP is used to efficiently estimate protein confidences (or quantities) from the directly measured peptide confidences (or abundances) [22]. The problem has previously been modeled as an NP-hard set cover problem [23], which weights proteins from a bipartite graph of proteins and peptides observed in a mass spectrometry experiment (with edge set denoted 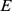). The previous method finds the smallest set of proteins that explain a certain amount of observed peptide evidence. Enforcing economy in the cardinality of the protein set prevents shared peptides, which may have come from several proteins, from incorrectly resulting in multiple protein identifications.

The QP relaxation minimizes the 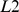-norm of the protein identifications (denoted 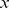); like the set cover formulation, this QP formulation enforces economy in the protein set. Also similar to the set cover formulation is the constraint requiring a certain quantity of peptides (weighted by their scores from the mass spectrometry experiment, each denoted 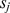) to be “explained.” The weighted number of explained peptides is given by the hyperparameter 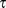. Each peptide (denoted 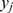) is further constrained to equal the sum of the proteins containing it (*i.e.* the sum of proteins adjacent to the peptide in the bipartite graph). Lastly, proteins are constrained to have nonnegative scores. The final QP is a projection of the zero vector onto a polytope:
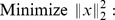





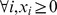
.
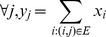


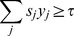



An initial feasible point was found by setting all 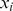 to a large value (in this case 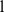, because the peptide scores 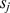 are approximate probabilities); this point was provided as a starting point for all agorithms. The hyperparameter was chosen as 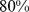 of all observed peptide scores 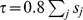.

Graphs were produced from two previously described mass spectrometry data sets [24], [25]: The first analyzes a small mixture of 48 purified proteins (plus common contaminants) searched against the human proteome (and a reversed database) as previously described. The second data set was aquired from yeast lysate.

Runtimes for optimizing the resulting QP was analyzed using the conic sampling method and the proprietary software package Mathematica (Table 0). Conic sampling runtimes were taken using UNIX user time, and Mathematica runtimes were taking using the built-in Timing command (so that the time to serialize the data into a native Mathematica format was not counted), and with no other processes running. In Mathematica the FindMinimum local optimization routine (which uses sparse vectors) was used, because the objective function is convex.

## Discussion

In the demonstrated examples, conic sampling algorithm performs comparably to or outperforms the primal affine-scaling and simplex algorithms. Particularly noticeable is the efficiency when applied to problems with many constraints, especially those with a great deal of sparsity. The efficiency of conic sampling on highly constrained LPs makes intuitive sense; each iteration of the conic sampling algorithm is 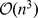 (before accounting for sparsity), and so the cost is roughly equivalent to 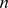 pivots with the simplex method (at a cost of 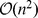 per pivot). When the number of expected pivots exceeds 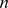, conic sampling may be much more efficient. Likewise, in the worst case (for a completely degenerate problem), each iteration of conic sampling will advance along an edge in a manner similar to Bland’s pivot rule. If this were the case, it would behave as an 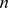-fold slower version of the simplex method. It should be noted that this 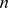-fold cost can be recovered by only *removing* the departed constraints from the Gram-Schmidt matrix (rather than rebuilding it from scratch); however, this can decrease the numeric stability of the algorithm by allowing errors to accumulate instead of resetting each time fixation is reached. Sparse Sherman-Morrison updating or sparse LU updating can achieve the same effect, but with increased numeric precision.

Furthermore, in LPs where the number of vertices is substantially higher than the dimensionality, the length of a greedy path along adjacent vertices (*i.e.* a path taken by the simplex methods) may become very large. On such highly constrained polytopes, a ray within the polyhedral cone defined by the objective vector and the bounding constraints has a high probability of arriving at a substantially improved vertex. If the improved vertices are sampled in a roughly uniform manner, then the expected number of iterations required by conic sampling will be logarithmic in the number of vertices. It may be possible to perform a more intelligent sampling of rays in the polyhedral cone (*i.e.* importance sampling), and yield a guaranteed expected runtime for certain families of polytopes. In this manner, conic sampling can be thought of as a generalization of simplex methods: rather than choosing an edge from a finite collection, the algorithm chooses a vector in a potentially infinite collection. Rules for choosing a vector from the polyhedral cone correspond to generalizations of simplex pivot rules.

Due to its simple geometric nature, the proposed method can be modified and applied to problems other than LP. On the family of QPs that compute projections onto polytopes, the conic sampling method is significantly more efficient than a sophisticated proprietary software package (Table 1); no doubt, a superior implementation (*e.g.* using the linear algebra library from Mathematica, Matlab, or LINPACK) could possibly be faster still, and could be applied to large, sparse projections, as well as to other QPs and convex optimization problems.

**Table 1:** QP runtimes from computational proteomics.

	Sigma 48	Yeast lysate
Variables (*n*)	392	3733
Constraints (*κ*)	393	3734

Method	Conicsampling	Mathematica	Conicsampling	Mathematica
	20.7942	20.7942	351.24	351.24
Runtime	**0.012**	1.41	**0.352**	145.68

The runtimes of Mathematica and the conic sampling algorithm (modified to compute the projection onto a polytope) are shown on QPs taken from computational proteomics (faster times are written in bold). Fully sparse vector data structures were used by conic sampling (sparse vectors are also used internally by Mathematica). The final objective value is presented using the default precision reported by Mathematica (both algorithms compute the same result).

The conic sampling algorithm, like several other existing methods, is motivated by a straightforward geometric notion; however, it does not suffer the same practical inefficiencies observed in the randomized simplex method. Randomness in LP solvers is typically used to avoid pathological behavior and is not responsible for good performance in practice. For conic sampling, the possibility of randomly jumping to a much improved vertex, and thus substantially narrowing the remaining vertices, is tightly intertwined with the algorithm’s performance. It should be noted that there may be deterministic variants of conic sampling that optimize over vectors in the polyhedral cone rather than choosing a random vector, and that these methods may still be faster in practice than the random approach.

The conic sampling method bears a resemblence to the gravity descent method. Both methods descend along facets and edges, permitting far more direct paths to the optimum; however, the gravity descent method solves a QP in order to decide which sets of constraints to abandon. In contrast, the conic sampling method takes a more restrictive, and less computationally expensive descent by projecting along the constraints of the polytope. This descent is not guaranteed to be the steepest, and will necessarily become immobile in at most 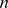 steps. Randomly sampling from a feasible ray not only allows the conic sampling method to become “unstuck” from the bounding constraints (without solving a QP), the manner with which it does so permits large jumps that move back through the strict interior, to a distal region of the convex hull.

The conic sampling method also benefits from largely ignoring unimportant constraints in a manner reminiscient of Seidel’s algorithm and the Matousek/Sharir/Welzl algorithm; however, rather than directly sifting through the constraints and finding those that bound the optimum, the conic sampling method implicitly does so. On polytopes with many facets not bounding the optimum, the chances of visiting these extraneous facets are very small, and once the objective function passes the greatest attainable on a feasible vertex of a facet, the facet is no longer considered.

The ease with which some geometrically motivated methods can be adapted to completely different convex optimization problems underscores its flexibility and generality. QPs and general convex optimization problems arise frequently, and form a superset of LPs. The greater mathematical complexity of these more general problems has resulted in fewer applicable algorithms and a greater difficulty in their optimization. Extensions of geometrically motivated methods like conic sampling algorithm may be of great use in solving these more general problems, especially in cases where the number of constraints dwarfs the dimensionality of the problem. Performing more intelligent random sampling in this cone may permit uniform sampling of the remaining volume or feasible vertices, and lead to algorithms with expected runtime bounds that are subexponential in the number of dimensions or constraints.
